# The Hippo signalling pathway maintains quiescence in *Drosophila* neural stem cells

**DOI:** 10.1038/ncomms10510

**Published:** 2016-01-29

**Authors:** Rouven Ding, Kevin Weynans, Torsten Bossing, Claudia S. Barros, Christian Berger

**Affiliations:** 1 Institute of Genetics, Johannes Gutenberg University, 55099 Mainz, Germany; 2 School of Biomedical and Healthcare Sciences, Plymouth University, PL4 8AA Plymouth, UK; 3 Peninsula School of Medicine, Plymouth University, PL6 8BU Plymouth, UK

## Abstract

Stem cells control their mitotic activity to decide whether to proliferate or to stay in quiescence. *Drosophila* neural stem cells (NSCs) are quiescent at early larval stages, when they are reactivated in response to metabolic changes. Here we report that cell-contact inhibition of growth through the canonical Hippo signalling pathway maintains NSC quiescence. Loss of the core kinases *hippo* or *warts* leads to premature nuclear localization of the transcriptional co-activator Yorkie and initiation of growth and proliferation in NSCs. Yorkie is necessary and sufficient for NSC reactivation, growth and proliferation. The Hippo pathway activity is modulated via inter-cellular transmembrane proteins Crumbs and Echinoid that are both expressed in a nutrient-dependent way in niche glial cells and NSCs. Loss of *crumbs* or *echinoid* in the niche only is sufficient to reactivate NSCs. Finally, we provide evidence that the Hippo pathway activity discriminates quiescent from non-quiescent NSCs in the *Drosophila* nervous system.

## Abstract (web summary)

*Drosophila* neural stem cells (NSCs) are quiescent at early larval stages but how this is regulated is unclear. Here, Ding *et al*. show that quiescence of NSCs is mediated by cell-contact inhibition via the Hippo pathway transmembrane proteins Crumbs and Echinoid, which in turn are regulated by nutrient levels.

## Graphical abstract

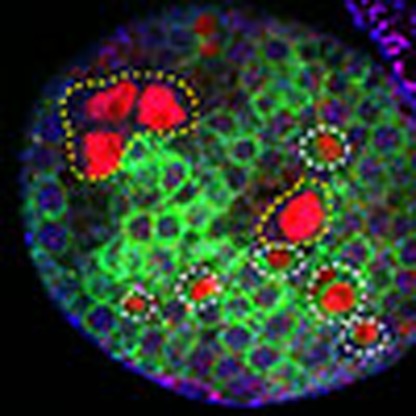

Stem cells are undifferentiated cells that have the unique ability to produce differentiating daughter cells and retain their identity by a process called self-renewal. Stem cells can exhibit a remarkable proliferative capacity, for example, during development or regenerative processes^1^,^2^. Deregulation of stem cell proliferation can lead to tumour formation or to a premature depletion of the progenitor pool^3^. Thus, stem cell proliferation has to be tightly regulated according to the cellular or organismal context. When proliferation is not required, stem cells are maintained in a state of quiescence in the G0-phase and need to be activated by systemic or local signals^3^,^4^. In *Drosophila*, neural stem cells (NSCs) proliferate in two phases^5^. The embryonic phase generates all cells of a functional larval central nervous system (CNS), while in the second proliferative phase cells forming the adult CNS are produced. In late embryonic stages NSCs enter quiescence, which requires intrinsic transcription factors^6^,^7^.

Changes in the physiological condition of the animal in response to feeding at early larval stages causes reactivation of NSCs^8^. The amino-acid sensing fat body releases systemic signals in response to the increase in dietary amino acids^8^,^9^ and CNS glial cells translate these signals into a local activating signal. They produce and secrete insulin-like peptides that activate the insulin/insulin-like growth factor signalling pathway in NSCs^10^,^11^. An initial step during reactivation is the drastic increase in NSC cell size from 4–5 μm during quiescence to 10–15 μm depending on the type of NSC^5^,^12^. Thus, growth in preparation for cell division is one of the initial hallmarks of NSC reactivation. The mechanisms regulating quiescence are less well understood. Glial cells secrete a glycoprotein (*anachronism*) that keeps NSCs in quiescence, but the precise molecular mechanism remains unknown^13^.

One of the major pathways that controls organ growth and cell proliferation in *Drosophila* and vertebrates is the conserved Salvador/Hippo/Warts signalling pathway (SHW)^14^,^15^,^16^. The SHW consists of a growth-repressive kinase cascade that modulates the activity of the transcriptional co-activator Yorkie (YAP/TAZ in vertebrates). The Hippo kinase activates the Warts kinase, which in turn directly phosphorylates Yorkie, creating a 14-3-3 binding site that restricts nuclear import and inactivates Yorkie^17^,^18^. If Hippo/Warts are inactive, non-phosphorylated Yorkie enters the nucleus and binds to transcription factors like Scalloped^19^,^20^ and activates its transcriptional program promoting cell growth and proliferation^21^,^22^. Numerous upstream regulators of the SHW have been identified, including cell–cell contact, the actin cytoskeleton, G-protein coupled receptors or planar and apico-basal cell polarity^23^.

In the vertebrate skin or the liver, de-repression of YAP has been shown to promote stem cell proliferation^24^. However, whether this is true in NSCs and whether changes in Yorkie/YAP activity are causative for altering growth and proliferation during normal CNS development remains unclear. In *Drosophila*, the SHW has been implicated in CNS development of the neuroepithelium and of glial cells in the optic lobe, and in cell growth of a specific population of glial cells (subperineural glial cells)^25^,^26^,^27^. However, for central brain NSCs, no function has been attributed to the SHW.

Here, we show that the SHW maintains quiescence of NSCs at the transition from embryo to larval life in *Drosophila*. Loss of the core kinases *hippo*/*warts*, or upstream regulators *kibra*/*Merlin*/*expanded*, leads to a premature initiation of cell growth and proliferation and thus reactivation from quiescence. Yorkie is inactive in quiescent NSCs and is necessary and sufficient for the reactivation and proliferation of NSCs. Cell–cell contact proteins Crumbs and Echinoid are expressed in both glial cells and NSCs and regulate the activity of Hippo and Warts, possibly via homophilic interactions in *trans*. The expression of Crumbs and Echinoid in glial cells and NSCs is nutrition-dependent, and their premature loss in glial cells is sufficient to initiate reactivation of NSCs. Moreover, the Yorkie activity discriminates between quiescent and non-quiescent NSCs, placing the SHW as a major regulator of growth in cellular quiescence in *Drosophila* NSCs.

## Results

### Loss of Hippo signalling causes premature NSCs reactivation

**Figure 1:**
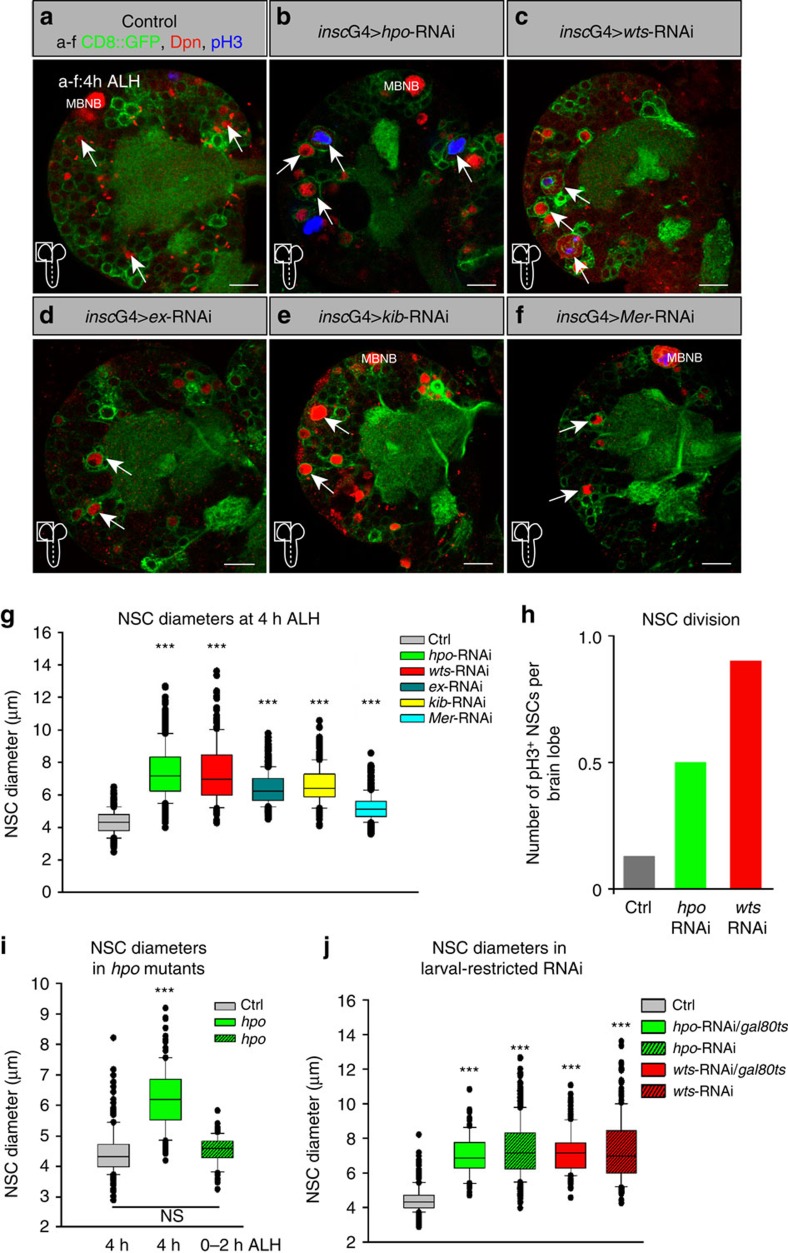
The SHW signalling pathway maintains quiescence in larval NSCs. (**a**) Wild-type larval brain lobe (*insc*G4> UAS-CD8::GFP, Control) 4 h ALH. Quiescent NSCs are small in cell size. NSCs of the mushroom body (MBNB) are big in cell size. No pH3-positive NSCs can be observed. (**b**-**f**) NSC-specific RNAi (*insc*G4> UAS-CD8::GFP and respective construct) of *hippo* (*hpo*)(**b**), *warts* (*wts*)(**c**), *expanded* (*ex*) (**d**), *kibra* (*kib*) (**e**) or *Merlin* (*Mer*) (**f**) leads to premature cell growth and cell division (observed in *hpo* and *wts*-RNAi) of NSCs. Arrows depict examples of NSCs. (**g**,**h**) Quantification of NSC cell diameters (**g**) and proliferation (**h**) in RNAi for Hippo pathway components. (**g**) ***P<0.001. Wilcoxon rank sum test. Median and s.d. were calculated from two biological replicates. Control *n*=355 NSCs (7 brain lobes); *insc*G4>*hpo*-RNAi, *n*=575 NSCs (11 brain lobes); *insc*G4>*wts*-RNAi, *n*=236 NSCs (5 brain lobes); *insc*G4>*expanded*-RNAi, *n*=400 NSCs (8 brain lobes); *insc*G4>*kibra*-RNAi, *n*=266 NSCs (5 brain lobes); *insc*G4>*Mer*-RNAi, *n*=506 NSCs (10 brain lobes); all 4 h ALH. (**h**) Number of NSCs in mitosis (pH3-positive) at 4 h ALH per brain lobe in wild-type (7 brain lobes) and *hpo*- and *wts*-RNAi (11 or 5 brain lobes). (**i**) Quantification of NSC cell diameters in *hpo*^*JM1*^/*hpo*^*KC202*^ (*hpo* in figure) trans-heterozygous mutants at 0–2 h ALH and 4 h ALH. ***P<0.001. Wilcoxon rank sum test. Median and s.d. were calculated from two biological replicates. Control 4 h ALH, *n*=150 NSCs (3 brain lobes); *hpo*^*JM1*^/*hpo*^*KC202*^ 0-2hmin ALH, *n*=65 NSCs (2 brain lobes); *hpo*^*JM1*^/*hpo*^*KC202*^ 4 h ALH, *n*=121 NSCs (3 brain lobes). (**j**) Quantification of NSC diameter of larval-restricted RNAi of *hpo* (*n*=120 NSCs in 8 brain lobes) and *wts* (*n*=172 NSCs in 10 brain lobes) in NSCs using the GAL80ts system. For comparison *hpo*- and *wts*-RNAi measurements without GAL80ts are added. All images are single focal planes, anterior up; Scale bar, 10 μm. NSC in red (Deadpan), CD8::GFP in green and phosphohistone H3 in blue (pH3). Pictograms denote the area of the brain shown in the picture. See also Supplementary Fig. 1.

To identify novel regulators of quiescence in NSCs, we depleted known growth regulators using RNAi-mediated gene knockdown in the *insc*-GAL4 pattern^28^. We scored NSCs (Deadpan-positive cells) cell size and proliferation rate (phosphohistone H3 (pH3)-positive NSCs) 4 h after larval hatching (ALH), when all NSCs are quiescent—small in cell size (∼4–5 μm) and non-proliferative (Fig. 1a,g,h)^5^,^10^,^12^. Exceptions are four NSCs of the mushroom body (MBNBs) and one ventrolateral NSC (lNSC) that do not enter quiescence, have a large cell diameter and constantly proliferate (Fig. 1a)^29^. These ‘non-quiescent NSCs' are quantified independently from all quiescent NSCs. First, we describe the fate of quiescent NSCs.

Knockdown of the core kinases of the SHW *hippo* or *warts* induces a marked premature increase in NSC cell size (Fig. 1b,c) from 4.5 μm (median, maximum 6.5 μm) in control brains 4 h ALH to 7 μm (median, maximum 13 μm; Fig. 1g). Since this suggests an early exit from quiescence, we tested for entry into S-phase using antibody staining for the S-phase cyclin CycE. We observed an increase in CycE-positive NSCs upon *warts*-RNAi (Supplementary Fig. 1a,b). Consequently, the number of pH3-positive mitotic NSCs was also significantly increased (Fig. 1h). Next, we examined the known upstream regulators of the SHW *expanded*, *kibra* or *Merlin* for their function in NSCs. Indeed, RNAi showed similar albeit less-pronounced effects and caused premature cell growth at 4 h ALH (Fig. 1d–g).

To ensure that this phenotype is not because of an impaired entry into quiescence, we analysed trans-heterozygous *hpo*^*JM1*^*/hpo*^*KC202*^ mutants^30^,^31^ at hatching (0–2 h ALH) and 4 h ALH (Supplementary Fig. 1c,d), and stage-17 embryonic brains of *wts*-RNAi (Supplementary Fig. 1e,f). In both situations NSCs did not show increased cell sizes or mitotic activity revealing a normal phase of quiescence (stage-17 at 0–2 h ALH). Interestingly, *hpo* mutant larvae exhibited a mild but significant increase in cell size at 4 h ALH mimicking the reactivation phenotype in *wts*-RNAi (Fig. 1i). Finally, we used the temperature-sensitive GAL4 repressor system GAL80ts to restrict the RNAi expression to only larval stages. Indeed, upon larval stage-restricted *wts*- or *hpo*-RNAi in NSCs we could monitor a similar increase in NSC diameter at 4 h ALH (Fig. 1j).

Thus, the SHW acts in *Drosophila* NSCs to maintain quiescence and cell-autonomous loss of pathway components leads to premature exit from quiescence.

### Yorkie relocates to the nucleus during reactivation

**Figure 2:**
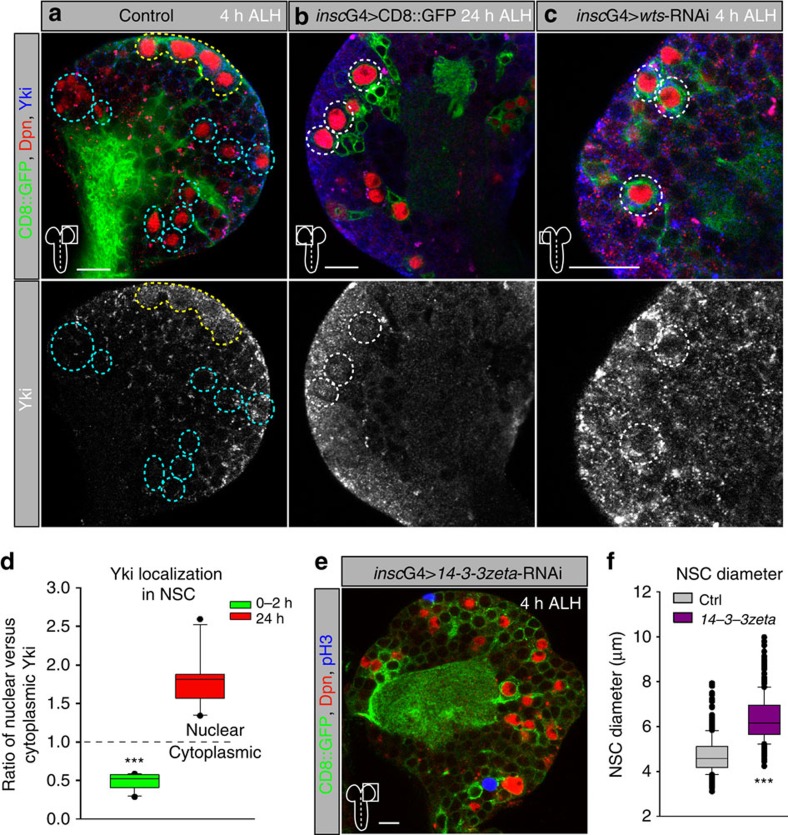
Yorkie is cytoplasmic during quiescence and re-localizes to the nucleus during reactivation of NSCs. (**a**-**c**) Subcellular localization of Yorkie in quiescent NSC at 4 h ALH (**a**, cyan circles, yellow circles show non-quiescent MBNBs), in reactivated NSCs at 24 h ALH (**b**, white circles) and in prematurely reactivated NSCs of *wts*-RNAi at 4 h ALH (**c**, white circles). Lower panels show only the Yorkie channel in monochrome. Circles depict examples of NSCs. (**d**) Quantification of the nuclear versus cytoplasmic localization of Yorkie in NSCs at 0–2 h (green, *n*=20, 5 brains from different animals) and 24 h (red, *n*=20, 5 brains from different animals) ALH. ***P<0.001. Wilcoxon rank sum test. (**e**) NSC-specific RNAi of *14-3-3zeta* leads to premature growth of NSCs at 4 h ALH. (**f**) Quantification of NSC cell diameters in wild-type and *14-3-3zeta*-RNAi at 4 h ALH. ***P<0.001. Wilcoxon rank sum test. Median and s.d. were calculated from two biological replicates. Control *n*=347 NSCs (7 brain lobes); *insc*G4>*14-3-3zeta*-RNAi, *n*=250 NSCs (5 brain lobes). All images are single focal planes, anterior up. Scale bar, 10 μm. See also Supplementary Fig. 2.

If the SHW maintains quiescence, the main effector Yorkie^32^ should be inactive and excluded from the nucleus in quiescent NSCs^17^,^18^, whereas we should observe nuclear localization in reactivated NSCs (24 h ALH). Antibody staining revealed no nuclear localization of Yorkie in quiescent NSCs (Fig. 2a,d and Supplementary Fig. 2). In contrast, at 24 h ALH a clear nuclear localization of Yorkie in reactivated NSCs can be detected (Fig. 2b,d and Supplementary Fig. 2). Since *wts*-RNAi caused premature reactivation 4 h ALH, we tested for premature nuclear localization of Yorkie and could monitor an increase in Yorkie protein levels and nuclear localization in NSCs that display a clear increase in cell diameter (Fig. 2c). Moreover, since phosphorylated Yorkie binds to 14-3-3 and stays inactive, we tested the loss of *14-3-3-zeta* with RNAi and observed premature growth of NSCs at 4 h ALH (Fig. 2e,f), presumably owing to early activity of Yorkie. Thus, Yorkie is inactive in NSCs during quiescence, and is activated and localizes to the nucleus during reactivation or upon *wts*-RNAi.

### Yorkie is necessary and sufficient for growth and proliferation

**Figure 3:**
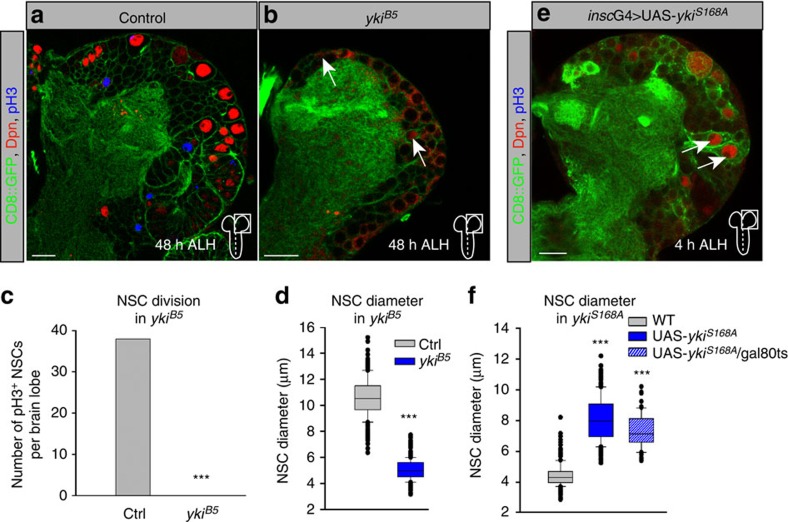
Yorkie is necessary and sufficient for NSCs growth and proliferation. (**a**,**b**) NSC growth and proliferation is impaired in *yki*^*B5*^ deletion mutants (**b**) compared with wild-type NSCs at 48 h ALH (**a**). (**c**) Average number of NSCs in mitosis (pH3-positive) at 48 h ALH in wild-type and *yki*^*B5*^ deletion mutants. (**d**) Quantification of NSC cell diameters *yki*^*B5*^ deletion mutants at 48 h ALH. ***P<0.001. Wilcoxon rank sum test. Median and s.d. were calculated from two biological replicates. Control *n*=240 NSCs (5 brain lobes); *yki*^*B5*^, *n*=459 NSCs (10 brain lobes). (**e**) Expression of *ykiS168A* in NSCs is sufficient to reactivate NSCs 4 h ALH. (**f**) Quantification of NSC cell diameters in ectopic expression of *insc*G4>*ykiS168A* (blue) or larval-restricted expression using the GAL80ts system (blue with white lines) at 4 h ALH. ***P<0.001. Wilcoxon rank sum test. Median and s.d. were calculated from two biological replicates. Control *n*=150 NSCs (3 brain lobes); *insc*G4>*ykiS168A, n*=200 NSCs (4 brain lobes); *insc*G4>*ykiS168A*; *tub*P*GAL80ts, n*=62 NSCs (4 brain lobes). All images are single focal planes, anterior up. Scale bar, 10 μm.

To determine whether Yorkie is also necessary for NSC growth and proliferation, we analysed *yki*^*B5*^ null mutants^32^. Homozygous *yki*^*B5*^ mutants are embryonically semi-lethal and most larvae die at approximately 48 h ALH. Whereas, wild-type NSCs at 48 h ALH have been reactivated and are highly proliferative (Fig. 3a,c,d), no reactivation of quiescent NSCs can be observed in the *yki*^*B5*^ mutants (no cell growth and no pH3-positive NSCs; Fig. 3b–d). Moreover, NSCs cell size and their mitotic index at 48 h ALH revealed that *yki*^*B5*^-mutant NSCs resemble quiescent NSCs (Fig. 3c,d). Next, we tested whether early expression of a constitutively active form of Yorkie (UAS-*yki*^*S168A*^)^17^ is sufficient to reactivate NSCs. Indeed, at 4 h ALH we observed a significant increase in NSCs cell diameter, which was also present when restricting the expression to only larval stages using the GAL80ts system (Fig. 3e,f). We conclude that Yorkie function is necessary and sufficient for NSCs reactivation and initiation of growth and proliferation.

### Yorkie activates the *bantam* microRNA during reactivation

**Figure 4:**
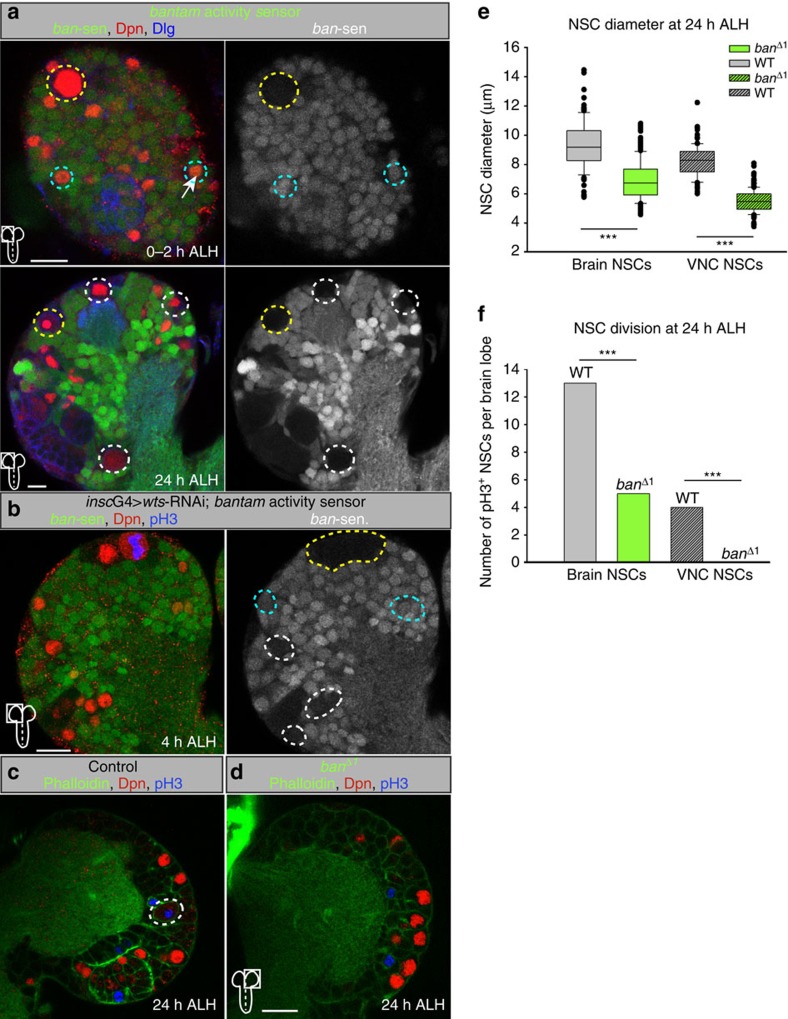
The microRNA *bantam* is active during reactivation and necessary for NSC growth and proliferation. (**a**) *bantam* (*ban*)-activity sensor in brain lobes at 0–2 h (upper panels) and 24 h (lower panels) ALH. No GFP signal monitors *ban* activity. Quiescent NSCs (cyan circles) do not have active *bantam* (GFP-positive), whereas reactivated NSCs (white circles) or the MBNB (yellow circles) have active *ban*. Right panels show GFP channel in monochrome. (**b**) NSC-specific *wts*-RNAi leads to premature *ban* activity (loss of GFP) at 4 h ALH. (**c**,**d**) NSC growth and proliferation is decreased in *banΔ*^*1*^ mutants (**d**) compared with control (**c**, white circles marks NSC in division) at 24 h ALH. Phalloidin in green. (**e**,**f**) Quantification of NSC cell diameters (**e**) and proliferation (**f**) in *banΔ*^*1*^ mutants at 24 h ALH. (**e**) ***P<0.001. Wilcoxon rank sum test. Median and s.d. were calculated from two biological replicates. Control brain *n*=109 NSCs (3 brain lobes); *banΔ*^*1*^ brain *n*=319 NSCs (7 brain lobes); control VNC *n*=109 NSCs (3 VNC); *banΔ*^*1*^ VNC *n*=253 NSCs (4 VNC). (**f**) Average number of NSCs in mitosis (pH3-positive) at 24 h ALH in wild-type of 3 brain lobes and 3 VNCs and *banΔ*^*1*^ 7 brain lobes and 4 VNCs. All images are single focal planes, anterior up. Scale bar, 10 μm. See also Supplementary Fig. 3.

Next we tested if the expression of the Yorkie target genes *four-jointed* (*fj*-lacZ)^33^, *expanded*^34^ and the microRNA *bantam*^35^ correlates with the subcellular translocation of Yorkie during reactivation. Fj-lacZ and expanded showed only weak expression in quiescent NSCs (4 h ALH) but clear upregulation at 48 h ALH (Supplementary Fig. 3a,b). We analysed the expression and activity of *bantam* that is known to regulate proliferation and growth^35^,^36^,^37^,^38^ by using a GFP-sensor system^36^. The loss of GFP expression and thus the activity of *bantam* coincides with the activation of Yorkie, as quiescent NSCs (4 h ALH) show strong GFP staining (Fig. 4a, upper panels) and reactivated NSCs (24 h ALH) have markedly reduced GFP signals monitoring *bantam* activity (Fig. 4a, lower panels). We combined the *bantam* sensor with *wts*-RNAi and examined an early activity of *bantam* at 4 h ALH (Fig. 4b), suggesting that premature reactivation by *wts*-RNAi expression causes early activation of the Yorkie downstream target *bantam*. To test if *bantam* is also necessary for NSC reactivation, we analysed *bantamΔ1* deletion mutants at 24 h ALH and observed NSC reactivation in the brain, but markedly reduced cell size and proliferative capacity (Fig. 4c–f). This effect seemed stronger in NSCs of the ventral nerve cord (VNC) at 24 h ALH, which in *bantamΔ1* mutants were indistinguishable from quiescent NSCs in control VNCs at 4 h ALH, with severely reduced cell sizes and nearly no pH3-positive NSCs (Fig. 4e,f). The difference between brain and VNC NSCs can be attributed to the spatiotemporal progression of NSC reactivation from anterior to posterior, which is also reflected in the size distribution of the wild-type control (Fig. 4e). Thus, we conclude that *bantam* is an important target of Yorkie during reactivation of NSCs, yet other unknown targets are likely involved in growth and proliferation of NSCs.

### SHW regulates reactivation depending on nutritional status

Because reactivation of NSCs is dependent on a nutritional stimulus and insulin signalling from CNS glia^8^,^10^,^11^, we tested if premature reactivation upon *wts*-RNAi depends on nutrition. Gene knockdown of *wts* in starved larvae resulted in a minor but still significant increase in cell size compared with *wts* knockdown in well-fed larvae (Supplementary Fig. 3c,d and Fig. 1c,g) but we could not detect pH3-positive NSCs. Thus, the SHW might regulate growth initiation of NSCs in parallel to the nutritional response model. This shows, that sensing of new nutritional resources occurs within the first 4 h ALH and SHW can initiate cell growth, but reactivation even in loss of the SHW depends on the nutritional status of the organism.

### Crumbs and Echinoid activate SHW during NSC quiescence

**Figure 5:**
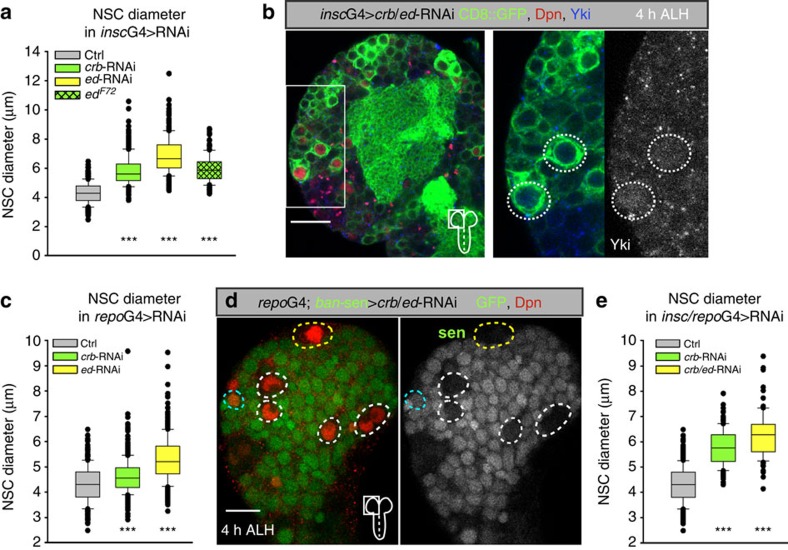
Crumbs and Echinoid are required in NSCs and glial cells in *cis* and in *trans* to activate SHW during quiescence. (**a**) Quantification of NSC cell diameters in NSC-specific RNAi (*insc*G4) of *crb* or *ed* and in the *ed*^*F72*^ mutant at 4 h ALH. ***P<0.001. Wilcoxon rank sum test. Median and s.d. were calculated from two biological replicates. Control brain *n*=355 NSCs (7 brain lobes); *insc*G4>*crb*-RNAi, *n*=313 NSCs (7 brain lobes); *insc*G4>*ed*-RNAi, *n*=300 NSCs (6 brain lobes); *ed*^*F72*^ *n*=1,169 (20 brain lobes). (**b**) NSC-specific *crb*/*ed* RNAi leads to premature nuclear Yorkie at 4 h ALH. Right panel shows a magnification of the marked area in the left panel. In the right panel only Yorkie (blue) and GFP (green) or Yorkie alone (monochrome) are shown. (**c**) Quantification of NSC cell diameters in Glia-specific RNAi (*repo*G4) of *crb* or *ed* at 4 h ALH. ***P<0.001. Wilcoxon rank sum test. Median and s.d. were calculated from two biological replicates. Control brain *n*=255 NSCs (5 brain lobes); *repo*G4>*crb*-RNAi, *n*=458 NSCs (9 brain lobes); *repo*G4>*ed*-RNAi, *n*=388 NSCs (7 brain lobes). (**d**) Glial-specific *crb*/*ed* RNAi leads to premature *bantam* activity (loss of GFP) in NSCs (red, Deadpan) at 4 h ALH. Right panel shows GFP (*ban*-sensor, monochrome) signal alone. (**e**) Quantification of NSC cell diameters in Glia- and NSC-specific RNAi (*insc*G4; *repo*G4) of *crb* or Crumbs*/ed* at 4 h ALH. ***P<0.001. Wilcoxon rank sum test. Median and s.d. were calculated from two biological replicates. Control brain *n*=145 NSCs (3 brain lobes); *insc*G4; *repo*G4>*crb*-RNAi, *n*=125 NSCs (3 brain lobes); *insc*G4; *repo*G4>*crb*/*ed*-RNAi, *n*=84 NSCs (2 brain lobes). All images are single focal planes, anterior up. Scale bar, 10 μm. See also Supplementary Fig. 4.

We sought to investigate how external signals regulated the cell-intrinsic, growth-repressing activity of the SHW during reactivation. Since a number of inter- and extracellular SHW regulators are known, we tested the transmembrane proteins Crumbs^39^,^40^,^41^,^42^ and Echinoid^43^,^44^ for their role in NSCs quiescence. When targeting *crb* or *ed* by RNAi in NSCs, a significant increase in NSCs cell size can be measured (Fig. 5a and Supplementary Fig. 4a,b) and in response to *crb/ed*-RNAi reactivated NSCs showed nuclear localization of Yorkie (Fig. 5b). For *ed* we analysed a embryonic/larval non-lethal hypomorphic allele (*ed*^*F72*^) that also exhibited premature reactivation revealed by increased NSC cell diameters (Fig. 5a) and incorporation of EdU monitoring S-phases (Supplementary Fig. 4c).

Since NSC reactivation involves niche glia cells^10^,^11^, we explored the role of niche signalling during quiescence. Using *repo*-GAL4 for glial-RNAi, we targeted *crb* or *ed* in glial cells only, which was sufficient to observe a similar significant but less-pronounced increase in NSC cell size compared with knockdown of *crb* or *ed* in NSCs (Fig. 5c and Supplementary Fig. 4d,e). To exclude that this is a consequence of altered SHW in glial cells, we analysed NSCs behaviour in glial *wts*-RNAi. In contrast to *crb*- or *ed*-RNAi we could observe a premature growth initiation in subperineural glial cells as described before (Supplementary Fig. 4f)^27^. The effect on reactivation of NSCs was stronger compared with glial *crb* or *ed* RNAi (Supplementary Fig. 4g). To test whether *wts*-RNAi and therefore premature glial growth can bypass the nutritional stimulus, we analysed the effect of nutritional deprivation. Similar to *wts*-RNAi in NSCs in nutrition-deprived conditions, *wts*-RNAi in glial cells in starved larvae leads to a minor reactivation phenotype and a less pronounced but still significant increase in NSCs cell diameter (Supplementary Fig. 4g). Thus, premature glial growth in *wts*-RNAi can initiate growth in NSCs but full reactivation is dependent on nutrition. Knockdown of *crb* or *ed* in glial cells did not result in a premature growth initiation in glial cells (compare Supplementary Fig. 4d,e with f). To show that the premature growth initiation in NSCs upon glial-RNAi of *crb* and/or *ed* is through altering the SHW pathway in NSCs, we made use of the *bantam* activity sensor. Indeed, we could monitor a premature activation of *bantam* (loss of GFP) in NSCs upon glial *crb/ed* RNAi (Fig. 5d). Therefore, knockdown of *crb* or *ed* in niche glial cells leads to premature reactivation of NSCs through altering Hippo activity in *trans*.

To test potential homophilic interactions of Crumbs^45^, we targeted *crb* simultaneously in both NSCs and glial cells by RNAi (*insc*-GAL4/ *repo*-GAL4) and observed an increase in the strength of premature reactivation of NSCs compared with glial knockdown alone (Fig. 5c,e and Supplementary Fig. 4h,i). Since the phenotype was similar to the NSC-specific RNAi of *crb* or *ed* alone we conclude that *crb* and *ed* act both in *trans* and in *cis*. Knockdown in glial cells removes the interaction in *trans* (between glial cells and NSCs) but leaves the interaction in *cis* (on NSCs), which causes a less-pronounced phenotype. Conversely, knockdown of *crb* or *ed* in NSC or in NSC and glial cells simultaneously interrupts both interactions in *cis* and in *trans* and causes stronger phenotypes.

Simultaneous glial- and NSC-specific knockdown of *crb* caused Yorkie nuclear localization in reactivated NSCs (Supplementary Fig. 4h) connecting *crb* function to the SHW. Finally, we assessed redundancy and performed double RNAi gene knockdowns of *crb* and *ed* simultaneously with the double driver GAL4 line (NSCs and glial cells concurrently), which caused NSC reactivation in the same degree as knockdown of *ed* alone (Fig. 5c,e and Supplementary Fig. 4i). Thus, other factors might compensate or influence the Hippo activity in NSCs along with *crb* or *ed*.

In conclusion, cell-contact inhibition of growth by niche glial cells through the SHW maintains quiescence in *Drosophila* NSCs. Interestingly, loss of Hippo signalling in the niche alone is able to initiate cell growth in NSC even during starvation.

### Glial Crumbs and Echinoid expression depends on nutrition

**Figure 6:**
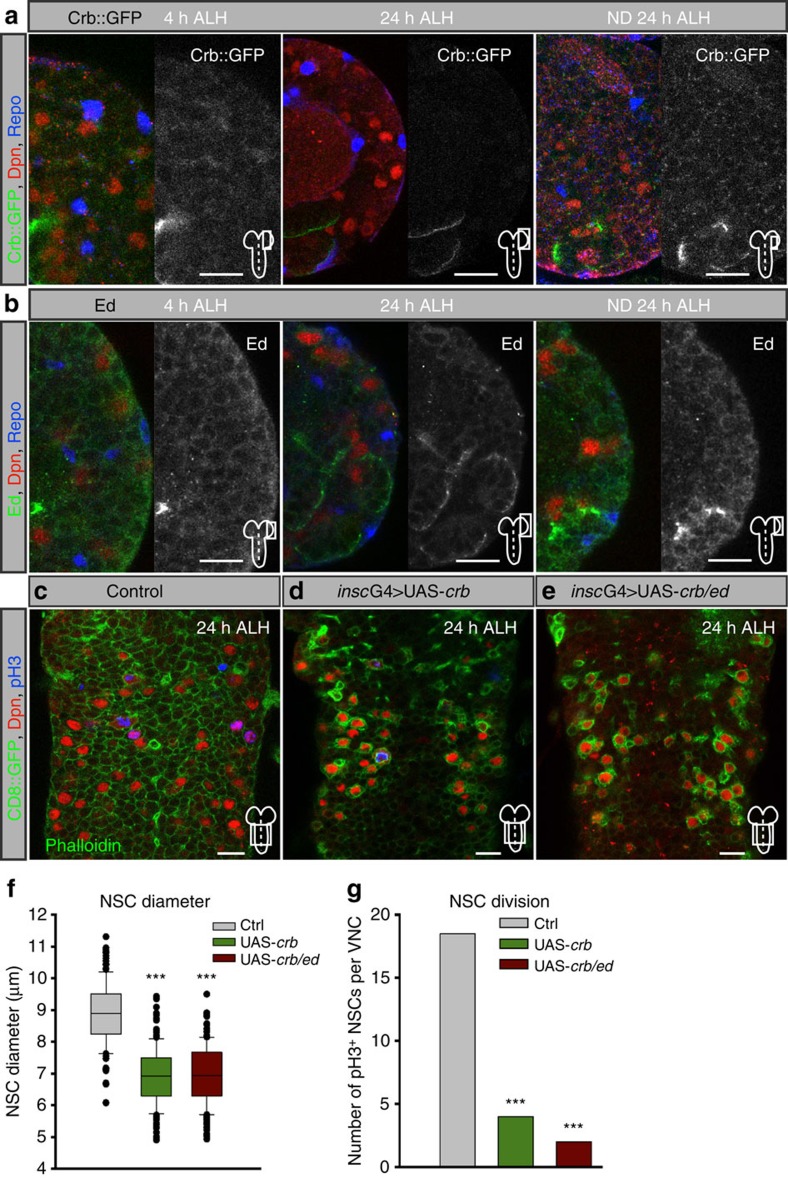
Crumbs and Echinoid are temporally expressed in glial cells and NSCs during quiescence and prolonged expression suppresses NSCs growth and proliferation. (**a**) Expression of Crumbs::GFP at 4, 24 and 24 h in nutrition-deprived (ND) ALH. Right side of each panel shows the GFP channel in monochrome. Crumbs expression is lost in both glial and NSCs after reactivation but persists in ND. (**b**) Expression of Echinoid at 4, 24 and 24 h in nutrition-deprived (ND) ALH. Right side of each panel shows the Echinoid channel in monochrome. Echinoid expression is lost in glial cells at 24 h ALH, while NSCs still express Echinoid, but persists in ND. (**c**-**e**) In comparison with wild type (**c**) prolonged expression in NSCs of *crb* (**d**) or *crb/ed* (**e**) leads to suppression of NSCs cell growth and division in ventral nerve cord NSCs at 24 h ALH. (**f**,**g**) Quantification of NSC cell diameters and mitosis upon NSC-specific prolonged expression of *crb* or *crb*/*ed* at 24 h ALH. (**f**) ***P<0.001. Wilcoxon rank sum test. Median and s.d. were calculated from two biological replicates. Control VNCs *n*=129 NSCs (3 VNCs); *insc*G4>UAS-*crb, n*=144 NSCs (3 VNCs); *insc*G4>UAS-*crb/ed, n*=165 NSCs (3 VNCs). (**g**) Ratio of NSCs in mitosis (pH3-positive) per counted VNCs at 24 h ALH in wild type of 3 VNC and *insc*G4>UAS-*crb* of 3 VNCs and *insc*G4>UAS-*crb*/*ed* of 3 VNCs. All images are single focal planes, anterior up. Scale bar, 10 μm. See also Supplementary Fig. 5.

Both Crumbs^46^ and Echinoid^47^ are expressed in epithelial cells and their role in glial cells and NSCs was surprising. Using a functional Crumbs::GFP fusion protein^48^, *crb*-mRNA *in situ* (Supplementary Fig. 5b) or antibody staining for Echinoid we observed expression of both in glial cells and NSCs during quiescence (Fig. 6a,b and Supplementary Fig. 5a–c). Since our data so far suggests a cell-contact inhibition of growth by niche glial cells we analysed whether Crumbs::GFP localizes to contact sites of glial cells and NSCs. Indeed, we could observe a slight accumulation in NSCs towards the contact site with glial cells (Supplementary Fig. 5d). To prove that NSC and glial cells indeed form cell–cell contacts, we stained for E-Cadherin and could monitor adherens junctions between glial cells and NSCs (Supplementary Fig. 5e). Crumbs::GFP expression in glial cells and NSCs was lost over time (8 and 24 h ALH, respectively) whereas Echinoid was downregulated mainly in glial cells (24 h, Fig. 6a,b and Supplementary Fig. 5a,c). Nutritional deprivation prolongs quiescence and Crumbs::GFP, *crb*-mRNA or Echinoid expression was maintained in glial cells and NSCs at 24 h ALH (Fig. 6a,b and Supplementary Fig. 5a,c,f), whereas early activation of the Insulin-like receptor signalling leads to premature loss of Crumbs::GFP in NSCs (Supplementary Fig. 5g). Thus, Crumbs and Echinoid are expressed in non-epithelial niche glial cells and NSCs during the phase of quiescence and are developmentally downregulated in response to nutrition.

### Ectopic *crb* causes decreased NSC growth and proliferation

Next we assessed whether ectopic expression of *crb* or *ed* would prolong quiescence. Expression of *crb* in glial cells leads to embryonic lethality, and its expression in brain NSCs results in cell clustering and an increase in NSCs of the MBNB (Supplementary Fig. 5h) complicating the analysis. These phenotypes were not apparent in NSCs of the VNC and we were able to analyse their growth behaviour and their mitotic index. Expression of *crb* was not able to suppress reactivation, but was sufficient to strongly reduce growth and proliferation at 24 h ALH (Fig. 6c–g). Co-expression of *crb* and *ed* had no additive effect on growth suppression, but the mitotic index was further reduced (Fig. 6e–g). Thus, although we observed a strong reduction in growth and proliferation, ectopic *crb* and *ed* did not extend quiescence, which might be owing to the nutritional reactivatory signal overcoming *crb* input.

### SHW discriminates between quiescent and non-quiescent NSCs

**Figure 7:**
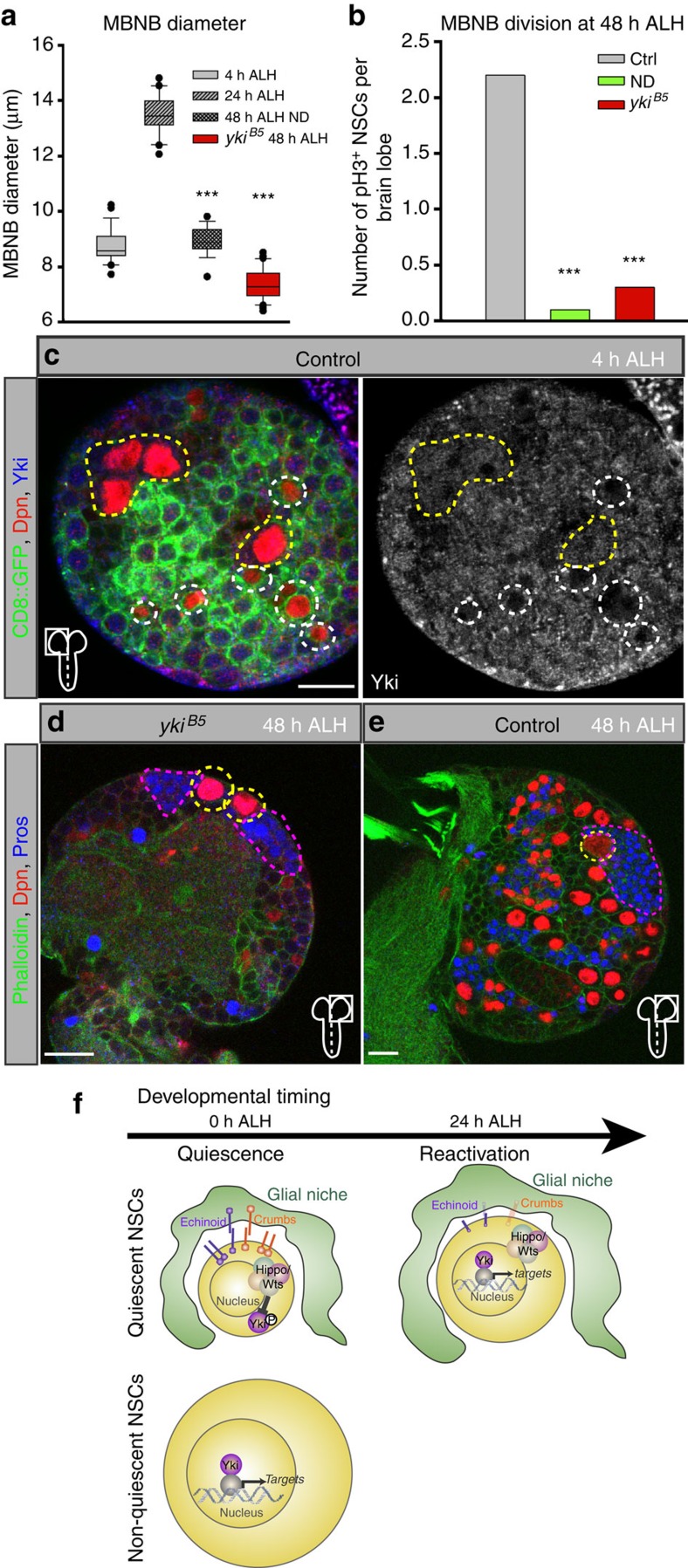
Yorkie activity discriminates between quiescent and non-quiescent NSCs. (**a**) Quantification of the cell diameter of NSCs of the mushroom bodies (MBNB) at 4, 24, 48 h in nutrition-deprived (ND) or *yki*^*B5*^ mutants at 48 h ALH. ***P<0.001. Wilcoxon rank sum test. Median and s.d. were calculated from two biological replicates. Control at 4 h *n*=28 NSCs (7 brain lobes); 24 h *n*=20 NSCs (5 brain lobes); 48 h ND *n*=19 NSCs (5 brain lobes); *yki*^*B5*^ at 48 h *n*=40 NSCs (10 brain lobes). (**b**) Quantification of MBNB in mitosis. Number of pH3-positive NSCs per brain lobes counted in 7 control brain lobes, 5 brain lobes in ND and 10 brain lobes in *yki*^*B5*^ mutants. (**c**) Yorkie is constantly nuclear in non-quiescent NSCs (yellow circles) and not nuclear in quiescent NSCs (cyan circles) at 4 h ALH. (**d**,**e**) NSCs of the MBNB in *yki*^*B5*^ (**d**) are smaller and have less Prospero-positive (Pros, blue encircled in magenta) progeny than compared with the control (**e**) at 48 h ALH. (**f**) Schematic of Crumbs- and Echinoid-mediated cell-contact inhibition of growth through the Hippo signalling pathway (upper panel). Homophilic interaction of Crumbs and Echinoid in *trans* on the glial niche and on the NSC inhibits Yorkie. During reactivation Crumbs and Echinoid are downregulated in response to nutrition in the niche, which inactivates the Hippo pathway and activates Yorkie. Yorkie is necessary and sufficient for reactivation, growth and proliferation. Non-quiescent NSCs have constant Yorkie activity (lower panel). All images are single focal planes, anterior up. Scale bar, 10 μm.

Next we sought to investigate whether the SHW discriminates quiescent from non-quiescent NSCs in *Drosophila*. Analysing the growth behaviour and mitotic index of non-quiescent NSCs of the mushroom bodies (MBNBs) at 4, 24 and 48 h ALH and in nutritional deprivation unravelled that their growth and proliferation depends on nutrition and requires active regulation (Fig. 7a,b). Testing the SHW we observed constant nuclear Yorkie levels in MBNBs (Fig. 7c) and expression of Fj-lacZ, expanded and activity of *bantam* (Fig. 4a and Supplementary Fig. 3a,b) confirming a continuous Yorkie activity. Indeed, in *yki*^*B5*^ mutants we could observe a lack of MBNBs growth and a marked reduction in the proliferative capacity of MBNBs at 48 h ALH (Fig. 7a,b,d) compared with wild-type MBNBs (Fig. 7e). We conclude that continuous Yorkie activity is necessary for MBNBs growth and proliferation and Yorkie activity discriminates between quiescent and non-quiescent NSCs in *Drosophila*.

## Discussion

NSCs need to tightly control the balance between proliferation and quiescence, since deregulation can lead to tumour formation or premature depletion of the progenitor pool^1^,^3^. In order to orchestrate their behaviour according to the status of the organism they need to communicate with their surrounding microenvironment. Similar to the neurogenic niche in vertebrates^49^, processes of glial cells in insects enwrap NSCs to form an enclosed chamber known as the trophospongium^50^. It is still debated whether *Drosophila* NSCs are independent of niche signalling and indeed NSCs in culture undergo asymmetric cell division to self-renew and produce differentiating progeny^51^,^52^, exhibiting very similar behaviour as their counterparts *in vivo*, like progressing through an intrinsically regulated series of transcription factors (temporal transcription factor cascade)^53^ and generating diverse cell types and lineages *in vitro* resembling the *in vivo* lineages in cell number and identity^54^. Conversely, loss of contact to the surrounding epithelium leads to a randomization of the mitotic spindle in isolated embryonic NSCs^55^ and compromising *Drosophila* E-Cadherin function in niche glial cells impairs the mitotic activity of NSCs^56^. During quiescence and reactivation NSCs show a strong dependency on extrinsic signals from glial cells. Glial cells express and secrete quiescence promoting factors^13^, or in response to nutrition, activating factors^10^,^11^. We now show that niche glial cells express transmembrane proteins Crumbs and Echinoid that act in *trans* to activate the SHW in NSCs to maintain quiescence and suppress cell growth. Thus, like vertebrate adult NSCs, *Drosophila* NSCs show different degrees of dependency on niche signalling. During quiescence both NSC populations need extrinsic cues from the niche to maintain quiescence (for example, this work^10^,^11^,^57^,^58^), whereas during the active-phase lineage progression maybe more cell-intrinsic, pre-programed and to a less degree depending on the niche^54^,^59^.

The SHW and its effector Yorkie/YAP have been widely implicated in stem cell biology and organ size restriction. In the vertebrate liver progenitors, the hepatocytes, the SHW controls quiescence of these stem cells^60^. Combined loss of Mst1/2 (homologues of *Drosophila* Hippo) resulted in loss of YAP phosphorylation, leading to a massive overgrowth and hepatocellular carcinoma. Other regenerative tissues, like the skin or the intestine, also harbour stem cells and an involvement of the SHW was likewise shown^61^,^62^,^63^,^64^,^65^. Our data now show that the SHW is also important for the regulation of quiescence in NSCs. The SHW is active during quiescence and suppresses inappropriate growth and proliferation of NSC in the *Drosophila* larvae. Similar to *Drosophila*, mouse adult quiescent NSCs (aNSCs) also show a prominent cell growth before the initiation of proliferation^59^,^66^. Thus, a similar mechanism of promoting quiescence by growth restriction might exist in adult vertebrate NSCs. Indeed, a recent molecular study on NSC quiescence showed that multiple SHW members like Lats2 (Warts homologue) or WWC2 (Kibra homologue) are upregulated in aNSCs, which after BMP4 exposure enter into a quiescence-like status in cell culture^57^. Moreover, our study demonstrates that a crosstalk between the niche glial cells and the stem cells via Crumbs and Echinoid activates the SHW to repress growth during quiescence. This might also be conserved in mouse aNSCs since Martynoga *et al*.^57^ showed that upon BMP4-induced quiescence expression of *crumbs2* (*CRB2*, *Drosophila crumbs* homologue) is also upregulated in aNSCs. Whether *CRB2* is expressed in the vertebrate niche and activates the SHW in NSCs during quiescence still has to be shown. Nevertheless, given these similarities it is attractive to speculate that the Hippo pathway might act as a general regulator of NSC quiescence in vertebrates and invertebrates.

In *Drosophila* two different populations of NSCs can be discriminated: NSCs that become significantly smaller and enter quiescence at the end of embryonic stages, and NSCs that constantly proliferate and do not go into quiescence. How this difference is established is not known up to now. Here we show that the activity of the transcriptional regulator Yorkie seems to be a major difference between these two populations of NSCs. Although quiescent NSCs have active Hippo signalling and thus no active Yorkie, the non-quiescent NSCs show constant nuclear Yorkie and constant expression of the known Yorkie-targets *bantam* and Four-jointed. We did not observe failure to enter into quiescence upon *wts*-RNAi or in trans-heterozygous *hpo* mutants and thus it seems less likely that SHW activation is needed to initiate quiescence.

Non-quiescent NSCs increase in size upon larval hatching and like in quiescent NSCs, this growth depends on nutrition and on Yorkie function. This also influences the proliferative capacity, since both populations of NSCs either showed no proliferation or exited proliferation prematurely. A similar function of YAP was described during vertebrate neurogenesis in the developing chick neural tube. YAP is highly expressed in NSCs and co-localizes with Sox2 a neural progenitor marker. Loss of YAP leads to premature differentiation, whereas overexpression leads to an increase in the progenitor pool and accelerated cell cycle progression^67^. More recently YAP expression was also found in the mouse ventricular zone in Sox2-positive progenitors^68^ and, importantly, simultaneous depletion of YAP and FAT4 or Dachs1 rescued a prolonged neuroprogenitor cell proliferation phenotype in a mouse model for Van Maldergem syndrome^69^. Therefore, Yorkie/YAP emerges as an important regulator of NSC biology and it is therefore of great importance to identify the precise molecular mechanisms and target genes by which Yorkie/YAP promote growth, proliferation and stem cell identity in NSCs. Interestingly, in *Drosophila* we could uncover an essential difference between NSCs in the brain and the VNC. In both populations of stem cells Yorkie activates its well-established target *bantam*. Yet, loss of *bantam* severely impaired growth and proliferation of the VNC NSCs, whereas the effects were milder in brain NSCs. Thus, to fully understand the function of Yorkie in NSCs it will be important in the future to unravel the stem cell-specific target genes that are regulated by Yorkie in NSCs.

## Methods

### Genetics

The RNAi fly strains obtained from the Vienna *Drosophila* RNAi Center (VDRC) are: *expanded*-RNAi (Stock number: 22994), *kibra*-RNAi (100765), *Merlin*-RNAi (7161), *crumbs*-RNAi (39177), *echinoid*-RNAi (104279), *hippo*-RNAi (104196), *warts*-RNAi (106174). The RNAi fly strains obtained from the Bloomington *Drosophila* Stock Center (BSC) are: *hippo*-RNAi (Stock number: 33614), *warts*-RNAi (34064), *crumbs*-RNAi (38373), *14-3-3zeta*-RNAi (31498). NSC-specific RNAi was performed with *insc*-GAL4 (w^1118^; P{GawB}*insc*MZ1407), glial-specific RNAi was performed with *repo*-GAL4 (w^1118^; P{GAL4}*repo*/TM6b, iab-lacZ). Both GAL4 driver lines carried UAS-*CD8::gfp* or UAS-*CD4td::gfp* and UAS-*dicer2*. For larval-restricted RNAi we combined the *insc*-GAL4, UAS-*CD8::gfp* with the *tub*P-*GAL80*[ts] (Bloomington Stock 7108) and embryonic phases were cultured at 18 °C before shifting to 29 °C just ALH. All other RNAi experiments were conducted at 29 °C during larval life—embryonic phases were cultured at 25 °C. Mutants alleles used were, *ed*^*F72*^, *hpo*^*JM1*^, *hpo*^*KC202*^, *yki*^*B5*^ and *banΔ*^*1*^ balanced over *CyO*, Pw[+mC]=Dfd-EYFP2, *CyO*, P(GAL4-*twi*.G)2.2, P(UAS-2xEGFP)AH2.2 or TM6B, Pw[+mC]=Dfd-EYFP3, *Sb*^*1*^ *Tb*^*1*^ *ca*^*1*^. UAS-*yki*^*S168A*^ (Stock number: 28818), UAS-*crb* (5544) and *fj*-LacZ (6370) were obtained from the BSC. The fly stock carrying the *crumbs*-extracellular domain tagged with GFP (Crumbs::GFP-A/TM3) was a kind gift from Yang Hong^48^. For nutritional deprivation egg collection were made on and hatched larvae transferred to agar plates prepared with 1 l PBS, 10 g agar, 50 g sucrose. For well-fed larvae collection, flies were reared on standard *Drosophila* fly food and egg collections were made on and hatched larvae transferred to agar plates prepared with 1 l apple juice, 27 g agar supplemented with dry yeast.

### Antibodies, *in situ* probes and immunohistochemistry

Immunohistochemistry experiments were performed as previously described^70^. In brief, larval CNSs were dissected in PBS, fixed in 4% formaldehyde. Washing steps were performed using PBS with 0.3% Triton X-100 (3 × 5 min) and primary antibodies were incubated overnight at 4 °C. Antibodies used were guinea pig anti-Deadpan (1:1,000, kind gift from Jürgen Knoblich), mouse anti-Pros (1:100, MR1A, Developmental Studies Hybridoma Bank, DSHB), mouse anti-Repo (1:100, 8D12, DSHB), mouse anti-Dlg (1:20, DSHB), mouse anti-pH3 (1:1,000, Cell Signaling Technology), mouse anti-β-Gal (1:375, Promega), mouse anti-Dig (1:1,000, Roche), anti-Echinoid (1:1,000, kind gift from Laura Nilson), rabbit anti-Yorkie (1:400, kind gift from Kenneth Irvine), rabbit anti-Expanded (1:1,000, kind gift from Richard Fehon).

*In situ* probe for *crumbs* was PCR-generated using the following forward primers: 5′-CGTTGGTGGCCAGAAATTGG-3′ and 5′-CACAGTGCTGACCCTCGAAT-3′ (5′-TAATACGACTCACTATAGGAGACCAC-3′) as reverse primer ((XX)=T7 sequence). After purification of the PCR product the *in vitro* transcription using T7 and the Dig RNA Labelling Mix (Roche) was used to generate the RNA *in situ* probe.

### EdU incorporation assay

To detect mitotic activity using EdU incorporation we dissected larval CNS at appropriate time points and incubated them for 1 h in 10 mM EdU/PBS. CNSs were fixed for 15 min in 4% formaldehyde/PBS and Alexa Fluor azide was detected according to the manufacturer's instructions (Click-iT EdU Imaging Kit, Invitrogen).

### Image acquisition and processing

Confocal images were acquired on a Leica SP5 confocal laser-scanning microscope using × 63 glycine immersion objective lens or a Leica SP8 confocal laser-scanning microscope using × 40 objective lens. All images represent single confocal sections except for images of EdU incorporation in *ed*^*F72*^ mutants that are projections of z-stacks. Images were processed using the Leica LAS, Adobe Photoshop and assembled in Adobe Illustrator. Cell size measurements and pH3-cell counts were done using the Leica LAS, statistical analysis were conducted using SigmaPlot. The medial cell body of NSCs were measured for cell diameter evaluation excluding the longest and the shortest axis of the cell.

### Intensity measurements for Yorkie localization

The Leica LAS AF software was used to determine the pixel intensities of each channel across a cell. The values for each channel were exported to Excel and the whole cell and nuclear areas were determined by the signal intensity of the Deadpan staining (nucleus) and the Phalloidin staining (outer cell membrane). The intersection was defined as cytoplasm. According to this definition the Yorkie signal intensity values were separated into nuclear and cytoplasmic fraction and pixel intensities were averaged for each fraction. The ratio between the means was calculated as a measure for the relative amount of protein per sub-compartment. SigmaPlot was used for statistical analysis.

## Additional information

**How to cite this article**: Ding, R. *et al*. The Hippo signalling pathway maintains quiescence in *Drosophila* neural stem cells. *Nat. Commun*. 7:10510 doi: 10.1038/ncomms10510 (2016).

## Supplementary Material

Supplementary Information Supplementary Figures 1-5
