# Supplementary material for: The Hippo signalling pathway maintains quiescence in Drosophila neural stem cells
Source: Nat Commun. 2016 Jan 29;7:10510. doi: 10.1038/ncomms10510 (PMC4740179; doi:10.1038/ncomms10510)
Supplement: Supplementary Information — Supplementary Figures 1-5 [file ncomms10510-s1.pdf]

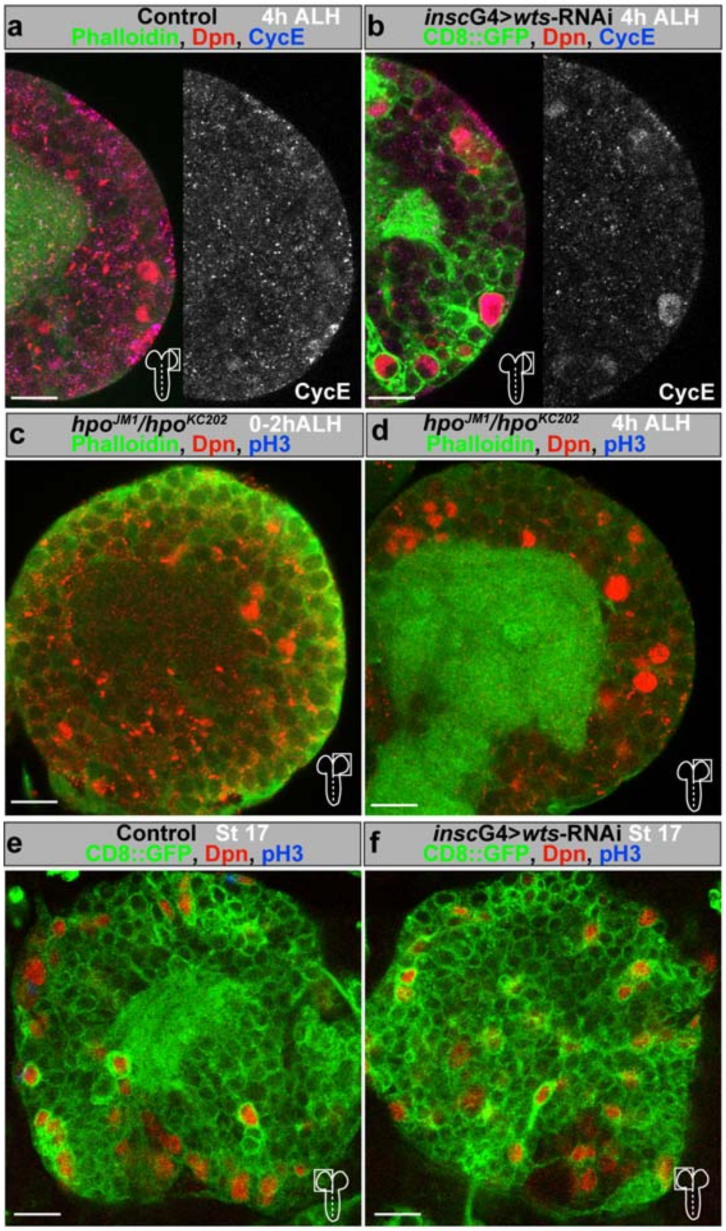

Supplementary Figure 1, related to Fig. 1 Upon *wts*-RNAi in NSCs CycE is upregulated and entry into quiescence is not impaired (**a,b**) The S-phase regulator CyclinE (CycE, blue) is weakly expressed in quiescent NSCs (**a**) but upregulated upon *wts*-RNAi in prematurely reactivated NSCs (**b**). Monochrome channel show only CycE staining. (**c**) Trans-heterozygous hpo^{JM1}/hpo^{KC202} at 0-2h ALH show no increase in NSCs cell size. (**d**) Trans-heterozygous hpo^{JM1}/hpo^{KC202} at 4h ALH show mild increase in NSCs cell diameter. (**e**) Control brain at embryonic stage 17 (St17). NSCs are small in cell size and do not proliferate. (**f**) Upon *wts*-

RNAi at 25°C no increase in cell size or mitosis can be observed in NSCs at embryonic stage 17. Entry into quiescence is not impaired.

All images are single confocal sections (if not stated otherwise) anterior up and scale bars represent 10µm. NSCs are labelled with Deadpan (Dpn, red), mitosis is marked by phosphohistone H3 (pH3, blue), either Gal4-driven CD8::GFP or phalloidin is in green. ALH, after larval hatching. Pictograms denote the area of the brain shown in the picture.

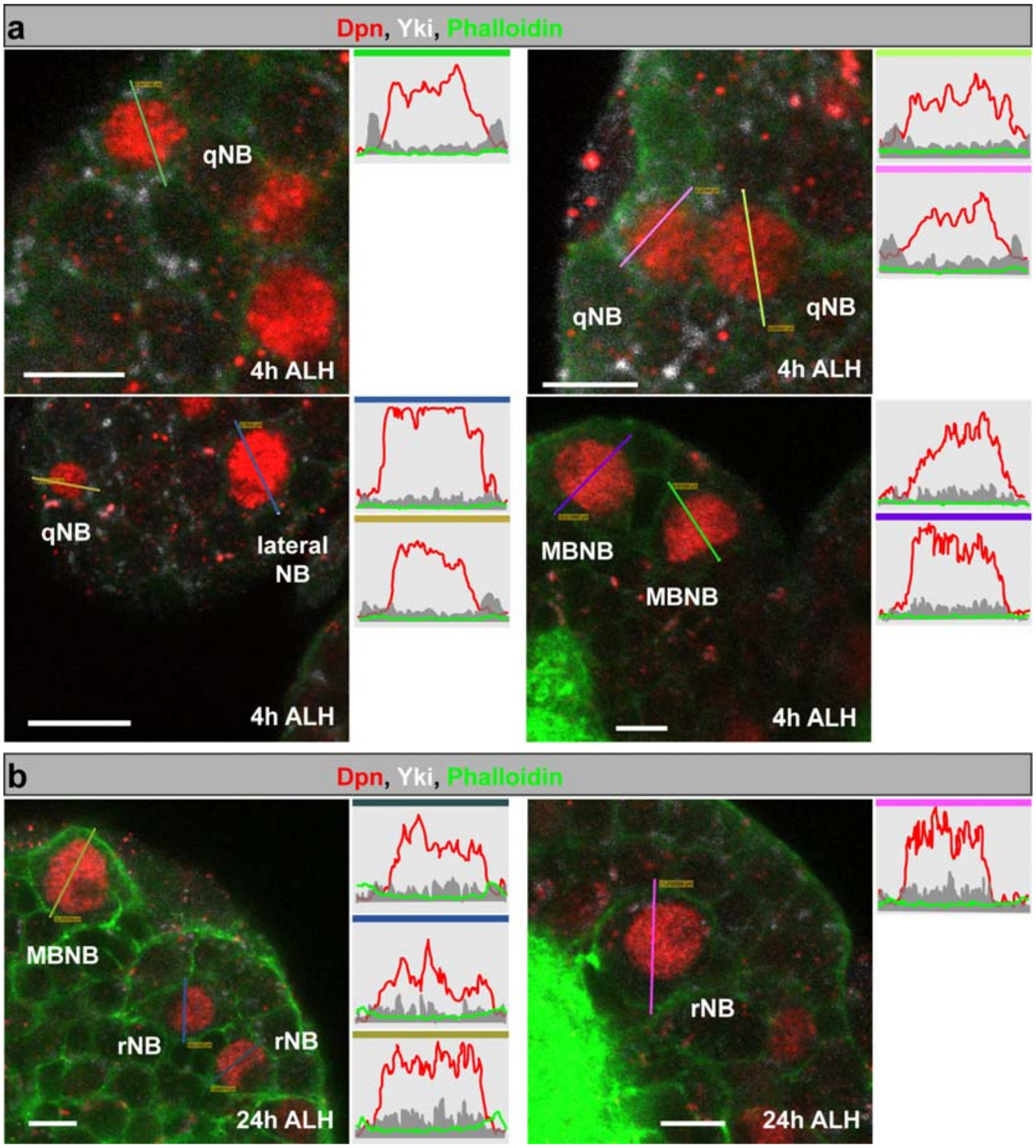

Supplementary Figure 2, related to Fig. 2 Quantification of the Yki nuclear and cytoplasmic signal in NSCs. (**a,b**) Representative images of measurements of Yki pixel intensities across the cell body (indicated line) of individual NSCs in quiescence (qNSC) or non-quiescent NSCs (lateral NBs and MBNB) at 4h (**a**) or reactivated NSCs (rNSC) at 24h ALH (**b**). The schematics to the right of each picture give the intensity measurement of the selected area (line) of each channel, red (Dpn, nucleus), green (Phalloidin, membranes) and dark grey (Yki). See methods for quantification procedure. All images are single confocal sections (if not stated otherwise) anterior up and scale bars represent 10µm.

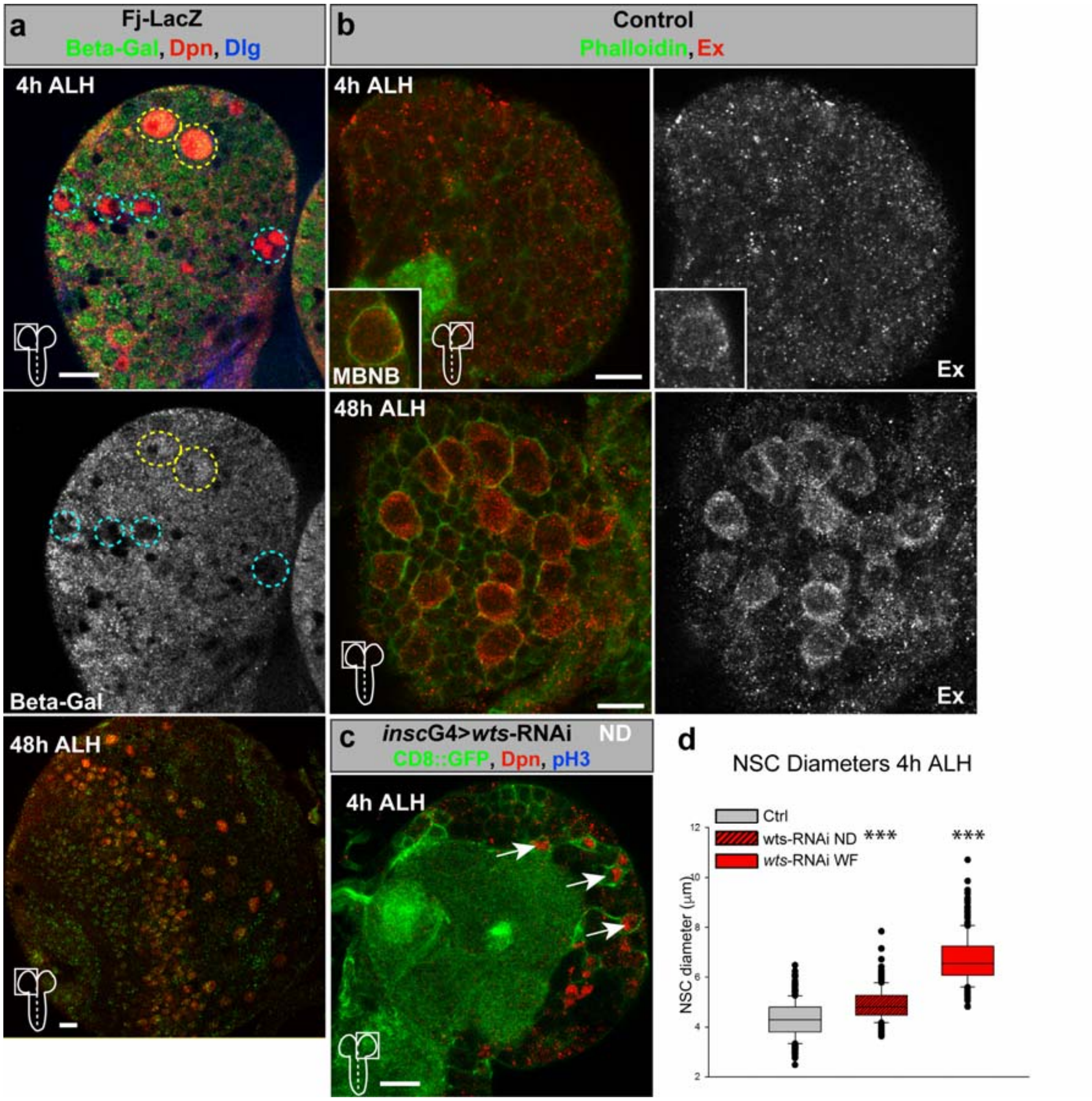

Supplementary Figure 3, related to Fig. 4 Yki targets Fj-lacZ and Ex are not expressed during quiescence but upregulated during reactivation, and premature reactivation upon *wts*-RNAi is dependent on nutrition. (**a**) Fj-lacZ is expressed in non-quiescent NSCs (yellow circles), but not in quiescent NSCs (cyan circles) 4h ALH. Middle panel shows ß-Gal channel in monochrome. Lower panel shows Fj-lacZ expression at 48h ALH. (**b**) Upper panel show Expanded (Ex) expression at 4h ALH. Only weak levels of Ex expression can be observed in the brain. Insert shows dorsally located NSC of the mushroom body with observable Ex expression. Lower panel show Ex upregulation in reactivated NSCs (big cells) at 48h ALH. Right panels show Ex channel in monochrome. (**c**) NSC-specific RNAi of *wts* in nutrition-deprived (ND) larvae leads to only minor reactivation phenotype. (**d**) Statistical analysis of NSC cell diameter at 4h upon *wts*-RNAi in well-fed (WF) or nutrition-deprived larvae. ***p

< 0.001. Wilcoxon rank sum test. Median and SD were calculated from two biological replicates. Control brain n= 355 NSCs (7 brain lobes); *insc*G4>*wts*-RNAi, ND n= 234 NSCs (5 brain lobes); *insc*G4>*wts*-RNAi, WF n= 290 NSCs (6 brain lobes). All images are single confocal sections (if not stated otherwise) anterior up and scale bars represent 10µm.

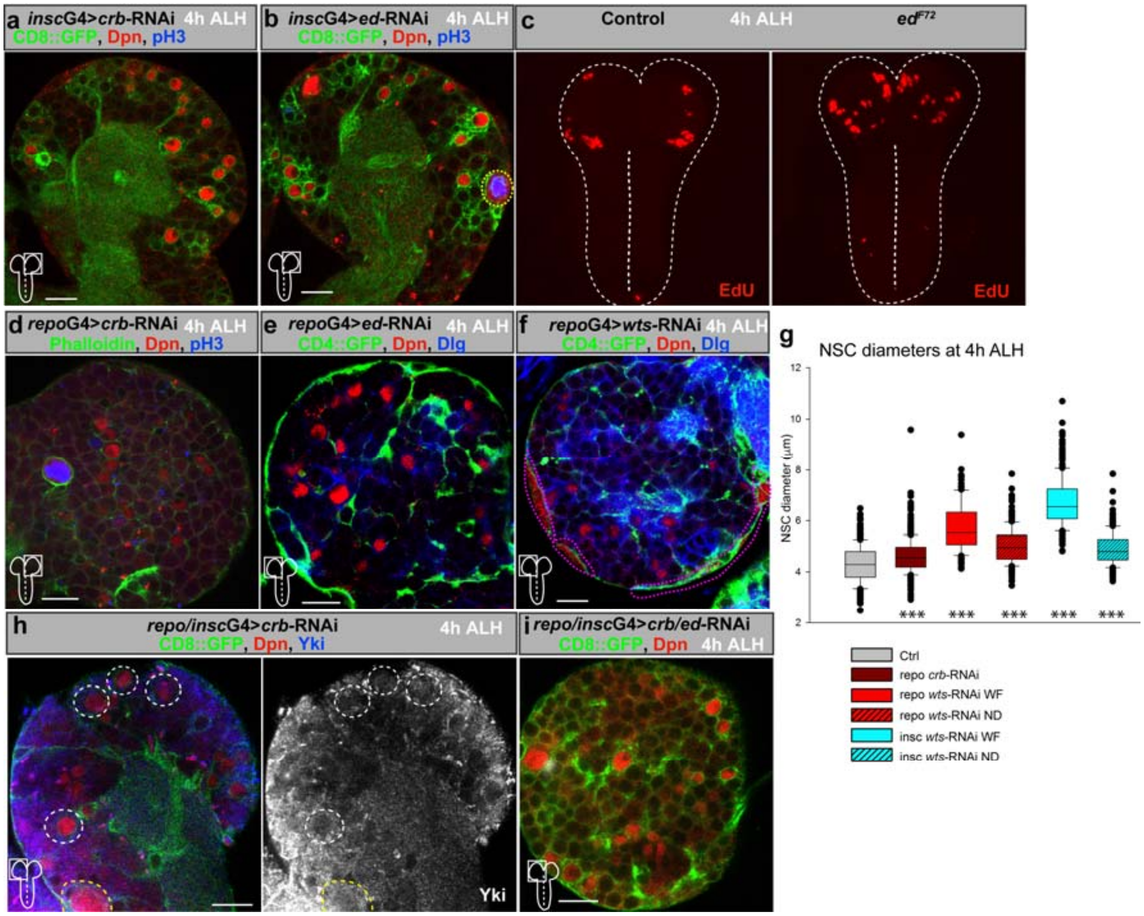

Supplementary Figure 4, related to Fig. 5 Crumbs and Echinoid are required in NSCs and glial cells in *cis* and in *trans* to activate SHW during quiescence. (**a**) NSC-specific (*insc*G4) RNAi of *crumbs* (*crb*) leads to increase in NSC cell diameter at 4h ALH. (**b**) NSC-specific RNAi of *echinoid* (*ed*) leads to increase in NSC cell diameter at 4h ALH (yellow encircled NSC shows non-quiescent lateral NSC). (**c**) EdU-incorporation (EdU, red) at 4h ALH is increased in ed^{F72} hypermorphic mutants (right panel) compared to control brains (left panel). (**d**) Glial-specific RNAi of *crb* leads to increase in NSC cell diameter at 4h ALH. Green shows Phalloidin staining. (**e**) Glial-specific RNAi of *ed* leads to increase in NSC cell diameter at 4h ALH. Green shows CD4td::GFP under the control of *repo*G4. (**f**) Glial-specific RNAi of *warts* (*wts*) leads to growth induction in subperineural glial cells (encircled in magenta) and increase in NSC cell diameter at 4h ALH. Green shows CD4td::GFP under the control of *repo*G4. (**g**) Statistical analysis of NSC cell diameter at 4h upon *crb*- or *wts*-RNAi in well-fed (WF) or nutrition-deprived (ND) larvae using *insc*G4 or *repo*G4. ***$p < 0.001$. Wilcoxon rank sum test. Median and SD were calculated from two biological replicates. Control brain n= 355 NSCs (7 brain lobes); *repo*G4>*crb*-RNAi, n= 458 NSCs (9 brain lobes); *repo*G4>*wts*-RNAi, WF n= 125 NSCs (3 brain lobes); *repo*G4>*wts*-RNAi, ND n= 225 NSCs (5 brain lobes); *insc*G4>*wts*-RNAi, WF n= 290 NSCs (6 brain lobes); *insc*G4>*wts*-RNAi, ND

n= 234 NSCs (5 brain lobes). (**h**) *crb*-RNAi simultaneously in NSCs and glial cells (*insc*G4, *repo*G4) leads to Yki nuclear localization in NSCs with increased cell diameter (encircled in white). The non-quiescent lateral NSC is encircled in yellow and show Yki nuclear localization. Right panel shows Yki channel in monochrome. (**i**) *crb*- and *ed*-RNAi simultaneously in NSCs and glial cells (*insc*G4, *repo*G4) leads to increase in NSCs cell diameter. All images are single confocal sections (if not stated otherwise) anterior up and scale bars represent 10µm.

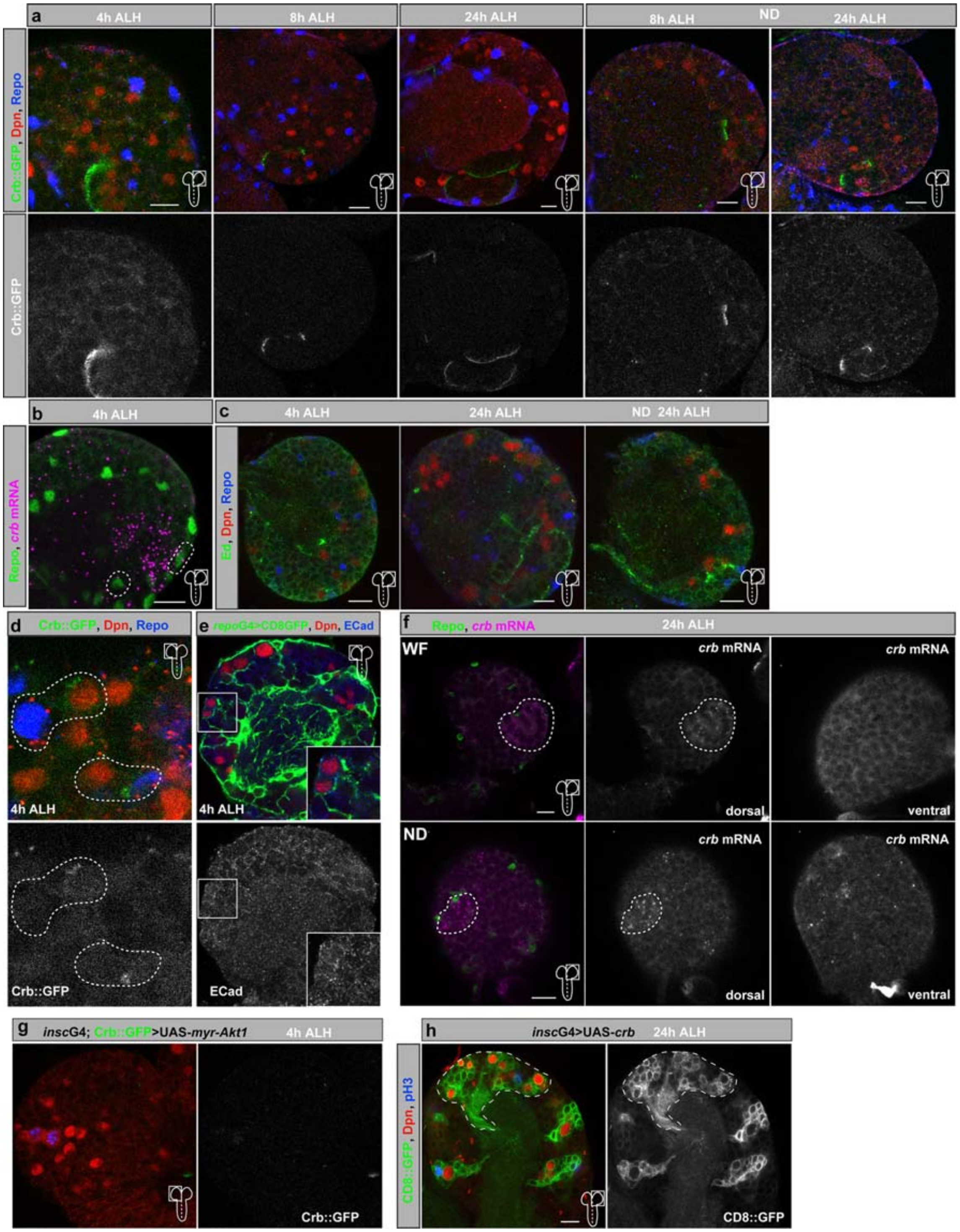

Supplementary Figure 5, related to Fig. 6 Crumbs and Echinoid are temporally expressed in glial cells and NSCs during quiescence and prolonged expression leads to extra Dpn-positive cells in the mushroom bodies. (**a**) Expression of Crb::GFP (fused to extracellular domain) in glial cells (Repo, blue) and NSCs (Dpn, red) at 4h, 8h and 24h or at 8h and 24h in nutritional deprivation (ND). Lower panel show GFP channel in monochrome. Crb expression is lost at 8h ALH from NSCs and glial cells, but persists under nutritional deprivation. (**b**)

Detection of *crb*-mRNA (magenta) using fluorescence *in-situ* hybridization in glial cells (Repo, green) Encircled in white are examples of glial cells. (**c**) Expression of Echinoid (Ed) in glial cells (Repo, blue) and NSCs (Dpn, red) at 4h and 24h or at 24h in nutritional deprivation (ND). (**d**) Crb::GFP accumulates at the interface of NSC and glial cell contacts (green and monochrome in lower panel, Crb::GFP; NSC labelled with Dpn in red). (**e**) E-Cadherin (blue and monochrome in lower panel, ECad) unravels adherens junctions between glial membranes (GFP, green) and NSCs (Dpn, red). (**f**) Expression of *crb*-mRNA is maintained in nutritional deprived conditions (lower panel) in comparison to well-fed conditions (WF, upper panel). Dorsal views show optic neuroepithelium (encircled in white) as internal control for *crb* expression. Ventral view shows maintained *crb*-expression. (**g**) Ectopic expression of myristoylated Akt1 (active form) in NSCs leads to premature reactivation of NSCs (Ph3 (blue) positive NSCs (Dpn, red) and early downregulation of Crb::GFP (green). Right panel shows Crb::GFP in monochrome. (**h**) Ectopic expression of *crb* in all NSC (*insc*G4) leads to increase in Dpn-positive cells in NSCs of the mushroom bodies (encircled in white) at 24h ALH. Left panel shows GFP signal in monochrome. All images are single confocal sections (if not stated otherwise) anterior up and scale bars represent 10µm.

#
